# Neonatal Piglet Temperature Changes: Effect of Intraperitoneal Warm Saline Injection

**DOI:** 10.3390/ani12101312

**Published:** 2022-05-20

**Authors:** Bryony S. Tucker, Kiro R. Petrovski, Roy N. Kirkwood

**Affiliations:** 1 School of Animal and Veterinary Sciences, University of Adelaide, Roseworthy, SA 5371, Australia; kiro.petrovski@adelaide.edu.au (K.R.P.); roy.kirkwood@adelaide.edu.au (R.N.K.); 2 Davies Livestock Research Centre, The University of Adelaide, Roseworthy, SA 5371, Australia

**Keywords:** rectal temperature, warming, birth weight, thermoregulation, temperature recovery, survival, intraperitoneal

## Abstract

**Simple Summary:**

The lighter a piglet is the worse is their ability to keep warm after birth. If they cannot recover from the reduction in temperature from in the womb to the crate they are at higher risk of hypothermia and death. A study was undertaken to investigate if providing them with a warm saline injection could improve their temperature over the first 24 h of life and their survival to weaning. Results show that lightweight piglets who received warmed saline had higher rectal temperatures at 24 h, increased colostrum intake, and greater survival at d 20.

**Abstract:**

Piglets are poor at thermoregulation immediately following birth and take up to 24 h to recover from their initial temperature drop. The present study aimed to determine if providing piglets with a 15 mL intraperitoneal injection of warm (45 °C) saline at birth would improve their internal temperature recovery to 24 h of age, and how the treatment interacted with birth weight (BWC = 1; ≤0.80 kg, BWC = 2; 0.81 kg to 1.10 kg, and BWC = 3; >1.10 kg), rectal temperature at 1.5 h (RC = 1; ≤32.0 °C, RC = 2; 32.10 °C to 35.0 °C, and RC = 3; ≥35.10 °C), and colostrum intake (CI = 1; <200 g and CI = 2, ≥200 g) to affect preweaning survival. Treated BWC1 piglets had improved rectal temperatures from 2 to 24 h. BWC3 piglets who consumed insufficient colostrum also had improved rectal temperature between 1 and 24 h post-birth. Colostrum intake was improved with saline injection in BWC2 piglets of RC1 and RC3 (*p* < 0.001) and BWC3-RC3 piglets (*p* < 0.001). Treated BWC1 improved survival to 20 d (*p* < 0.001). Irrespective of BWC, piglets from all RC had greater survival when injected with saline. The greatest difference was for piglets in RC1, likely due to all BWC1 piglets falling within this category. The results suggest that an intraperitoneal injection of warmed saline is an effective way to improve piglet temperature recovery to 24 h, colostrum intake, and survival in low-birth-weight piglets. These findings will be helpful for producers who have large numbers of low-birth-weight piglets born and are able to provide individual care.

## 1. Introduction

Piglets are born with relatively little adipose tissue and no brown fat, meaning they have a limited energy supply to thermoregulate and for mobility to obtain colostrum [1]. They experience a sharp decline and subsequent slow recovery in body temperature over the first 24 h of life [2,3,4]. The temperature decline can impair their ability to move and thus access colostrum and milk, resulting in hypoglycaemia and chilling, predisposing them to death by overlay or starvation [5,6].

A piglet’s initial temperature decline and its ability to thermoregulate are directly related to its weight [3]. Smaller piglets have a higher surface area to volume ratio and proportionally greater heat loss, and so are at greater risk of mortality due to hypothermia [7].

The ambient temperature in the farrowing location is usually set for the sows’ thermal requirements [8,9], which are below the piglet lower critical temperature [1] and so piglets must rely on additional heat sources such as heat lamps and/or mats. However, this heated area is stationary and is located away from the udder, with the total distance depending on the sow [10]. In order to maintain body temperature as well as having to adapt to the temperature change outside of the heated area, the movement back and forth by the piglets to obtain access to colostrum/milk uses some of their energy. For piglets that have very little energy due to their size and/or missed previous milk letdowns, this can be critical to their survival.

Previous studies that have suggested intervention strategies such as supplementary nutritional products (e.g., milk from other sows or cows, or energy-rich supplements) had varying results but all reduced the available stomach capacity for colostrum consumption [11,12,13]. Further, studies have shown the drying of piglets to have some effect on short-term temperature improvement, but few have shown long-term benefit to survival, suggesting minimal effects on the piglet internal temperature [4,7,14,15]. Despite data from previous studies, the success seemed to be heavily dependent on the piglet’s morphology, the quality of its environment, and the method of external or internal intervention.

In humans and companion animals, hypothermia is often treated with warm saline combined with warming of the skin surface [16,17,18]. Additionally, it is standard practice to use fluids warmed to 37–41 °C to prevent pre- and intra-operative hypothermia in patients [19]. Some safety studies in dogs have showed that intravenous (IV) fluid at both 40 °C and 65 °C was a safe and effective means of treating hypothermia [17,18]. Administration of warm IV fluids could be an alternative to providing a heat source that is not stationary and will not reduce stomach space. However, in commercial practice, the administering of IV fluids is neither practical nor economically viable. Therefore, we hypothesized that giving a bolus intraperitoneal injection of saline warmed to 45 °C, being a similar but higher temperature than their in utero environment [20,21], into potentially compromised piglets would ameliorate body heat loss and improve preweaning piglet survival.

## 2. Materials and Methods

This experiment was performed in September and October 2021, at the University of Adelaide, Roseworthy piggery, SA, approved by the institutional Animal Ethics Committee (Approval number; S-2021-046).

### 2.1. Housing and Management

The raw data set included 104 piglets (55 male and 49 female) born to 11 multiparous sows (Large white × Landrace; parity 2 to 4; 2.95 ± 0.49) born within observation hours. Total born litter sizes were recorded, including born alive and stillborn but excluding mummified piglets.

Sows were moved into one of three farrowing rooms at about 110 d of gestation and housed in conventional individual farrowing crates (0.5 m × 2 m) equipped with sow and piglet level nipple drinkers. The farrowing house was environmentally controlled and was maintained at about 22 °C throughout lactation using an automatic thermostat system and cool cells. Farrowing crates were equipped with a heat mat on one side of the sow set on a heating curve from 31 °C at farrowing to 24 °C prior to weaning. Crates were scraped daily, and health and welfare checks performed a minimum of 2 times daily throughout the study. From entrance to the farrowing house until farrowing, sows were fed a standard wheat/barely/lupin-based lactation diet formulated to supply all necessary nutrients (14 MJ DE/kg, 17% protein, and 0.8% SID lysine) at 2.5 kg/d. Once farrowed, sows were fed to appetite via hand feeding twice per day until weaning. Sows were induced to farrow by vulva injection of 125 µg cloprostenol (Juramate^®^, Jurox Pty limited, Rutherford, New South Wales, Australia) at 0700 and 1300 h on d 114 of gestation [22]. Farrowing observations were conducted daily from 0630 to 1700 h and piglets received an iron injection and a Toltrazuril drench (Baycox^®^, Elanco Australasia Pty Ltd., Macquarie Park, New South Wales, Australia) at 4 d of age.

**Figure 1:**
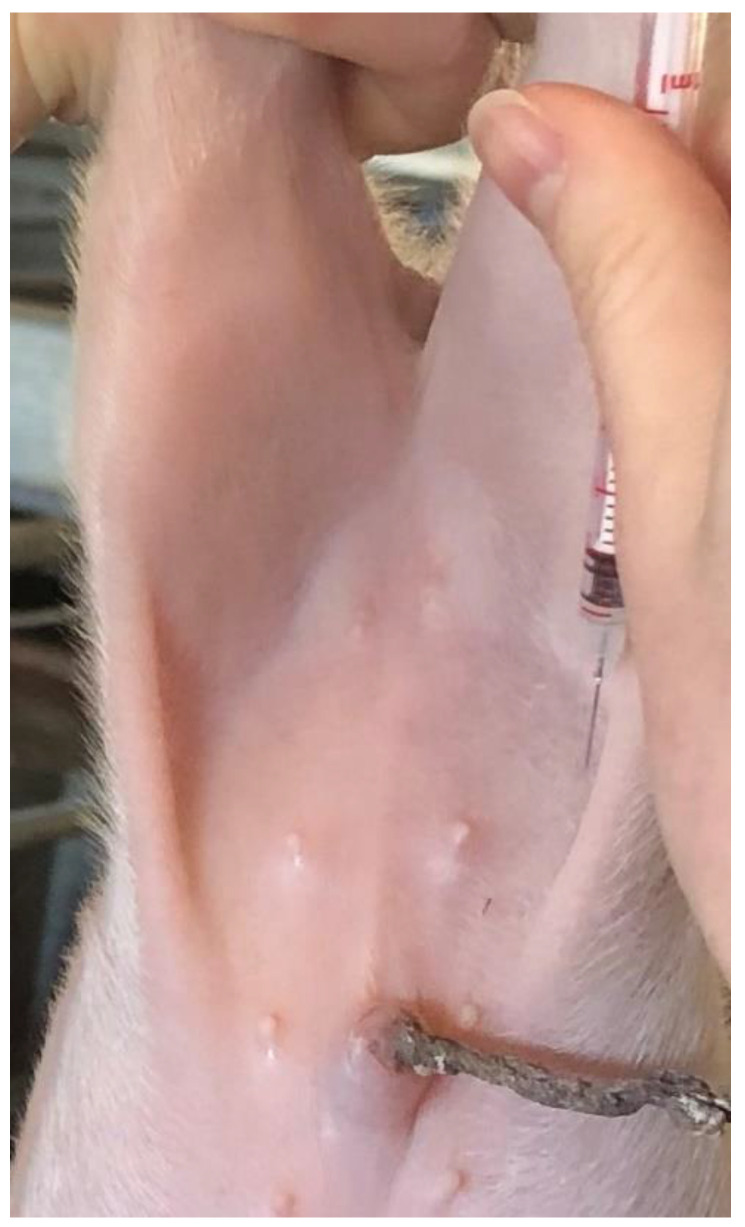
Intraperitoneal injection site of piglets.

Piglets were assigned prenatally by their birth order to receive 15 mL intraperitoneal saline warmed to 45 °C (saline, *n* = 52) or to receive no treatment (control, *n* = 52). The saline dose was based on the author’s clinical experience. Saline was warmed using bottle warmers (NUK, Zeven, Germany) and the temperatures monitored using digital meat thermometers. All piglets were held head down by their rear legs and saline piglets injected as shown in Figure 1 using a 20 mL syringe and a 20 g − ½ inch needle.

At expulsion of each piglet, the birth time and the rectal temperature of the sow were recorded as representative of the piglet birth temperature. At 0.25 h after birth, the piglets were weighed and received their pre-allocated treatment. The rectal temperatures of the piglets were recorded 2 min post saline injection or 2–4 min from picking up (control pigs) and at 1, 1.5, 2, 4, and 24 h relative to their birth time. At 4 and 20 d, piglet rectal temperatures were recorded again. Piglet weights at 24 h and at 20 d were used to calculate suckling weight gain. Colostrum intakes (*CI*) were calculated from 15 min and 24 h piglet weights using the equation developed by Devillers et al. [23]:

(1) $$CI=-217.4+0.217\times t+\text{1,861,019}\times \frac{W}{t}+BW\times \left(\frac{54.8-\text{1,861,019}}{t}\right)\times (0.9985-3.7\times 10^{-7}\times t_{fs}^{2})$$

where *CI* = colostrum intake (g), *W* = piglet body weight at 24 h (kg), *BW* = piglet body weight at birth (kg), *t* = age (min), and *t_fs_* = time elapsed from birth to first sucking (min). On the basis of the research of Devillers et al. [23], t_fs_ is assumed to be 30 min and *t* is 1440 min.

Colostrum intake was categorized into 2 levels on the basis of 200 g being the recommended minimum amount of colostrum needed to survive: level 1 includes piglets who consumed <200 g of colostrum and level 2 includes piglets who consumed ≥200 g. Survival was recorded to 20 d of lactation.

### 2.2. Statistics

All statistical analyses were performed with SAS version 9.4 (Statistical Analysis Software, Cary, NC, USA). For the statistical analyses, data were bootstrapped at root of 24 using PROC SURVEYSELECT resulting in a total 1589 observations at piglet level and 142 sow-level observations available for the analyses. The accuracy of the data was tested using PROC MEANS with all means of the bootstrapped data being similar to the original data to the second decimal point.

Correlations between rectal temperature and time were tested using PROC CORR with the output being the Pearson’s correlation coefficient and the respective 95% confidence intervals. Correlation was considered to be very high if *r^2^* ≥ 0.90, high if *r^2^* = 0.7 to 0.89, moderate if *r^2^* = 0.5 to 0.69, low if *r^2^* = 0.3 to 0.49, and negligible if *r^2^* < 0.3 [24]. Rectal temperature and body weight across time were moderately correlated (*r^2^* = 0.60), thus, prior to analysis, some data were manipulated:

Piglet birth weights were assigned to one of three categories: low (BWC = 1; ≤0.80 kg), mid (BWC = 2; 0.81 kg to 1.10 kg), and high (BWC = 3; >1.10 kg). The 0.8 kg value is based on commercial experience indicating pigs of this weight or less have minimal survival and documented evidence that piglets of 1.1 kg or heavier had good survival rates [24]. Piglet rectal temperatures at 1.5 h after birth were assigned to one of three categories (RC): low (RC = 1; ≤32.0 °C), mid (RC = 2; 32.1 °C to 35.0 °C), and high (RC = 3; ≥35.1 °C). The 1.5 h time was chosen, as it is a time point at which the piglets should start to show temperature recovery, thus indicating their vitality. Further, limitations from the human rectal thermometer restricted recording piglet temperatures below 32 °C, resulting in likely biased data prior to this time point. Significance was set at the *p* < 0.05 level.

The effect of the birth weight, time of measure relative to birth, treatment, parity and colostrum intake, and the interactions between birth weight, time of measure, treatment, and colostrum intake, on the rectal temperature of piglets were estimated using a Mixed model in PROC MIXED, as presented in Equation (2):

(2) $$\begin{array}{l} \mathrm{temperature}=[\mathrm{birth}\;\mathrm{weight}\;\mathrm{category}\;+\;\mathrm{time}\;\mathrm{of}\;\mathrm{measure}\;\mathrm{related}\;\mathrm{to}\;\mathrm{birth}\;+\;\mathrm{treatment}\;\mathrm{group}\;+\;\mathrm{parity}\\ \;\;\;\;\;\;+\mathrm{colostrum}\;\mathrm{group}\;+(\mathrm{birth}\;\mathrm{weight}\;\mathrm{category}\;*\;\mathrm{time}\;\mathrm{of}\;\mathrm{measure}\;\mathrm{related}\;\mathrm{to}\;\mathrm{birth}\\ \;\;\;\;\;\;*\;\mathrm{treatment}\;\mathrm{group}\;*\;\mathrm{colostrum}\;\mathrm{group}){]}_{\mathrm{piglet}\;\mathrm{ID}} \end{array}$$

where parity was a random factor and piglet ID was treated as a repeated measure. The preliminary model also tested the effects of litter size, farrowing room and piglet sex, but these were found to be not significant as confounders (*p* > 0.1).

The outputs of all PROC MIXED models were least-square means, their respective standard errors, and differences between least-square means.

The effect of the interaction between birth weight, treatment, and temperature category on colostrum intake was estimated using a Mixed model in PROC MIXED, as presented in Equation (3):

(3) Colostrum intake=(birth weight category∗treatment group∗rectal temperature category)

The preliminary model also tested the effects of sow, parity, sex, litter size, and farrowing room, but these were found to be not significant as confounders (*p* > 0.1).

The effect of the interaction of body weight category, treatment, and colostrum intake grouping on piglet suckling weight gain was estimated using a Mixed model in PROC MIXED, as presented in Equation (4):

(4) $$Suckling\;weight\;gain\;\left(kg\right)={\left(birth\;weight\;category*treatment\;group*colostrum\;group\right)}_{P}$$

where *p* = sow parity. The preliminary model also tested the effects of sow, sex, litter size, and farrowing room, but these were found to be not significant as confounders (*p* > 0.1).

The effect of birth weight category, and interaction of sex and treatment group with birth weight category on survival to 20 d were estimated using linear regression in PROC GLIMMIX (binary distribution), as presented in Equation (5):

(5) $$\begin{array}{l} \mathrm{survival}=\mathrm{birth}\;\mathrm{weight}\;{\mathrm{category}}_{\mathrm{fh},\mathrm{ls}}\;+\;(\mathrm{birth}\;\mathrm{weight}\;\mathrm{category}\;*\;\mathrm{treatment}\;\mathrm{group}{)}_{\mathrm{fh},\mathrm{ls}}\\ \;\;\;\;\;+(\mathrm{birth}\;\mathrm{weight}\;\mathrm{category}\;*\;\mathrm{sex}{)}_{\mathrm{fh},\mathrm{ls}}+\;(\mathrm{birth}\;\mathrm{weight}\;\mathrm{category}\;*\;\mathrm{colostrum}\;\mathrm{group}{)}_{\mathrm{fh},\mathrm{ls}} \end{array}$$

where *fh*= farrowing room and *ls*= litter size. The preliminary model also tested the effects of sow and parity, and treatment group and sex alone, but these were found to be not significant as confounders (*p* > 0.1). The outputs of all PROC GLIMMIX models were the geometric means and their respective 95% confidence intervals.

The effect of the interaction of birth weight category, sex, and treatment group with rectal temperature category on survival to 20 d were estimated using linear regression in PROC GLIMMIX as presented in Equation (6):

(6) survival=(birth weight category∗RC)+(sex∗RC)+(treatment group∗RC)+(colostrum group∗RC)

where *RC*= rectal temperature category.

## 3. Results

### 3.1. Temperature

**Figure 2:**
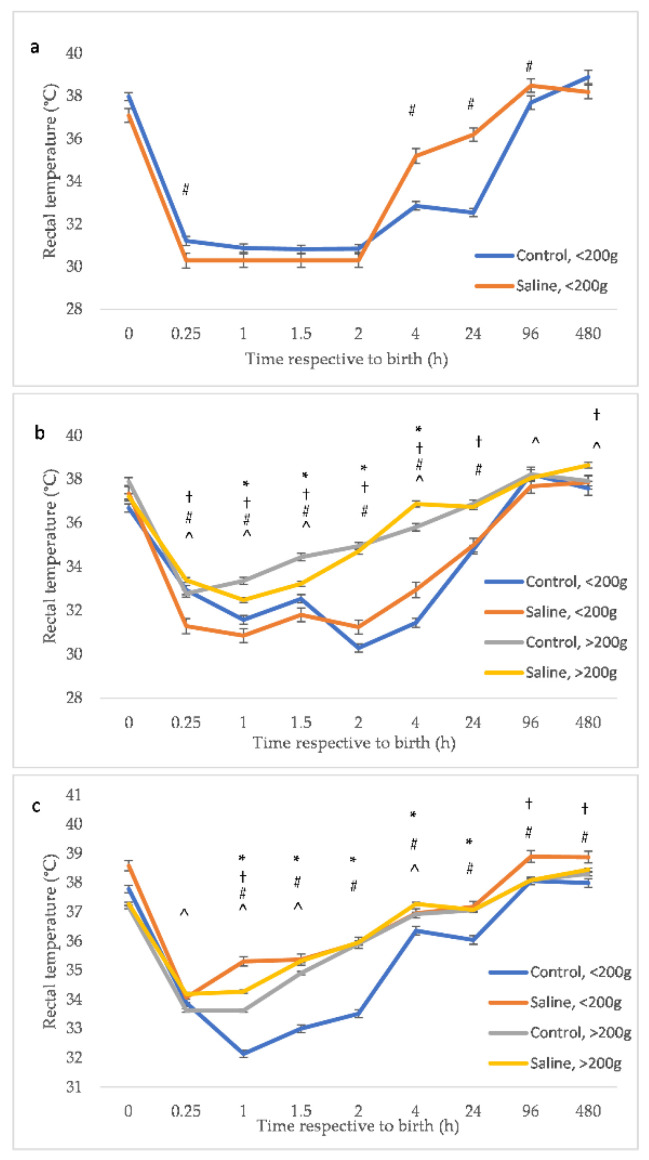
Mean ± standard error for the piglet rectal temperature for 13,330 piglet observations after bootstrapping at a root of 24, from birth to 20 days (480 h) within bodyweight category. Figure 2a presents BWC1 (includes piglets with birth weights ≤0.8 kg). Figure 2b presents BWC2 (includes piglets with birth weights 0.81–1.1 kg). Figure 2c presents BWC3 (includes piglets with birth weights ≥1.2 kg). Piglets were control or received a saline injection and ingested less than or greater than 200 g of colostrum. Adjusted for parity. * Denotes a significant (*p* < 0.05) effect of colostrum consumption within control piglets control piglets. † Denotes a significant effect of colostrum intake within saline piglets. # Denotes a significant difference between treatment groups for piglets consuming <200 g colostrum. ^ Denotes a significant treatment effect for piglets consuming >200 g colostrum.

Intraperitoneal injection of saline warmed to 45 °C increased the rectal temperature of BWC1 piglets from 2 h (*p* < 0.001; Figure 2a). BWC2 and BWC3 piglets showed an increased rectal temperature at time points between 0.25 h and 4 h from birth (BWC2 = 1, 1.5 and 4 h; *p* < 0.001; BWC3 = 0.25, 1, 1.5, 2 and 4 h; *p* < 0.001; Figure 2b,c).

### 3.2. Colostrum Intake

Medium weight piglets within RC1 and RC3 increased their colostrum intake when provided with warm saline (*p* < 0.001; Table 1). Heavier piglets showed a significant decrease in colostrum intake when in RC1 but an increase in the RC3 (*p* < 0.001). Unlike heavier piglets, lightweight piglets did not show a significant increase in colostrum intake either in the lowest (*p* = 0.27) or highest (*p* = 0.77) RC, due to large standard errors, although numerically they showed the same pattern of improvement as in the lowest rectal temperature category.

### 3.3. Suckling Weight Gain

Lightweight piglets consumed less than 200 g and, if treated, had lower weight gains than control piglets (*p* < 0.001; Table 2). The opposite was evident for medium-weight piglets consuming sufficient colostrum, with treated piglets having increased weight gain (*p* < 0.001). Heavier piglets were not influenced by treatment regardless of colostrum intake (*p* = 0.61 and 0.27, respectively).

### 3.4. Survival

Table 3 lists the survival of piglets to day 20 by BWC across treatment, sex, and colostrum intake. The BWC1 piglets injected with warm saline had an improved survival at 20 days from 11.7 to 100% despite none consuming more than 200 g of colostrum (*p* < 0.001; Table 3). Sex indicated that females had lower survival than males particularly in BWC1. BWC2 piglets showed decreased survival with increased colostrum intake but BWC3 piglets showed the opposite.

Table 4 lists the survival of piglets to day 20 by RC across BWC treatment, sex and colostrum intake category. No lightweight piglets (BWC1) had rectal temperatures within the medium (RC2) and high range (RC3) at 1.5 h relative to birth (Table 4). Piglets that were injected with saline had greater survival at 20 days than all control piglets across relative temperature groups (Table 4). Female piglets with RC 1 survived less than male piglets from the same RC.

We also noted that when modelling colostrum intake by treatment group in BWC1 piglets, mean colostrum intake was increased with the saline injection (control = 92.9 ± 3.4 g; saline = 189.0 ± 5.7 g). This presumably influenced their relative survivals.

## 4. Discussion

Thermoregulation is critical for newborn piglets; however, they have little energy reserves to rely on. Current mainstream strategies can enhance energy loss, limit colostrum intake, and/or have only short-term benefits [11,15,21]. Consequently, our objective was to test a method, based on treatment of hypothermic humans and companion animals, to maintain and or improve piglet internal temperature in the critical period after birth. Our data show that when administered saline was warmed to 45 °C, rectal temperatures in all BWC piglets were improved at certain critical time points in succession. Further, piglets receiving warm saline had improved colostrum intake and preweaning survival.

Injecting warm saline resulted in an increase in rectal temperature in BWC1 piglets at 2 h to 24 h after birth despite no BWC1 piglets consuming >200 g of colostrum, which is common in production. However, the 200 g recommended minimum average colostrum intake is based on the ‘average’ piglet. It is reasonable to suggest that the minimum would be lower in lower birth weight piglets and the 189 g consumed by treated BWC1 pigs was entirely adequate. It should also be mentioned that this performance indicator is reliant on a calculation based off of temperature and weight, both factors in the analysis [23]. Although this method is widely accepted and referred to it has the potential to increase error in these situations. We were unable to determine if there was an improvement from 0.25 h to prior to 2 h, as piglet temperatures dropped below 32 °C which was the minimum reading for the digital thermometer. BWC2 piglets who consumed <200 g colostrum had improved rectal temperature between 2 and 4 h when injected, whilst piglets who consumed >200 g colostrum had a lower temperature from 1 to 1.5 h but higher at 4 h and 20 d compared to the non-injected BWC2 piglets. These results show how critical colostrum consumption was and that if sufficient colostrum was consumed by medium-sized piglets, then there was less need for internal temperature intervention. However, BWC3 consistently had lower rectal temperatures in piglets who consumed <200 g colostrum and received no saline injection than all other BWC3 piglets. This suggests that if BWC3 piglets who consumed <200 g colostrum were provided with a warm saline injection, their rectal temperature could be sustained at a similar level to heavy piglets who consumed >200 g colostrum. Overall, these results show that for the more at-risk lightweight piglets, provision of a warm saline injection can increase rectal temperatures over time, and for larger piglets there was a greater colostrum–temperature interaction, which could be manipulated by providing this internal intervention.

As rectal temperatures increased so did colostrum intakes across BWC. Further, colostrum intake was greater in BWC2 piglets when provided the saline injection, as it was also in BWC3 piglets in RC3. Treated BWC1 piglets showed a numerically increased mean colostrum intake but the standard errors were larger, particularly those in RC1. This may be due to a subset of piglets within the low BWC, with a lower potential for survival regardless of reasonable intervention, which would be identifiable by other measures suggested in previous studies but not considered in this present study design [25,26]. Therefore, injecting warmed saline had not only improved rectal temperature but serendipitously also colostrum intake and/or potential for intake. Suckling weight gain was significantly higher in BWC1 and BWC2 piglets who did not receive the saline injection; however, the mean suckling weight gain for all piglets was within reasonable production standards. Therefore, injecting warm saline did not have a negative effect on growth to d 20. It could be suggested that the stress from additional handling and injecting could have disadvantaged the piglets, contributing to this lower weight gain. However, as no sham injection treatment group was used, this cannot be confirmed or denied.

The improvements in temperature resulted in markedly increased survival for the lightest piglets at 20 d and also for BWC3 piglets. Further, treatment increased survival across all RC but especially for low temperature piglets. Since saline is an easily absorbed fluid, it could be argued that this increase may also involve a potential rehydration effect, aiding their survival. However, in a preliminary study, we tested the effect of intraperitoneal injection of saline at about 39 °C, which we assume to represent hydration alone, on survival to 9 d which resulted in higher mortality rates with 52 of 67 non-treated piglets and 34 of 62 treated piglets surviving (Tucker et al., unpublished data). Further, in the present study, the authors noted no clinical signs of dehydration. Although we acknowledge that the effect of hydration cannot be separated within this study design, findings of the preliminary study support our theory of the effect being predominantly a result of a temperature effect on the piglets.

Other studies have documented a similar increase in rectal temperature with interventions such as drying and warming under heat lamps. However, those studies did not show the same sustained improvement in survival unless environmental temperatures were far from ideal [4,15]. Our data showed an increase in survival in piglets from all rectal temperature categories at 1.5 h when injected with warm saline. We infer that this increase across body weight and rectal temperature categories suggests a greater sustained temperature improvement, reducing the amount of energy the piglets are required to mobilize, thus maintaining their critical stores and improving the rate of survival. This was further supported by the increased colostrum intake across BWC and RC, most notably in BWC1 piglets.

Colostrum is the key to having sufficient energy for thermoregulation and survival and growth [27,28]. Providing piglets with fresh colostrum directly by collection and feeding or assisting onto the teat to suckle is a direct way of ensuring colostrum intake and thus subsequent survival [29,30]. However, both collection and assisting are time consuming and difficult. As no easy and efficient collection method of colostrum collection from sows exists, such practices are rarely applied on farm. Alternatively, energy supplements are quick and easily accessible, often provided as a drench or easily administered via pump bottle as a supplement to suckling [13,31]. However, energy supplements can increase energy available for absorption of the piglet but reduce the available stomach space for consumption of colostrum, which is the goal of providing assistance to piglets for improved survival [11,13]. Therefore, assuming appropriate training, our internal intervention with warmed saline, which is simple and straight forward, to directly improve temperature whilst indirectly improving colostrum intake, may have higher potential for application in production.

Localized environmental (heat lamp) and external interventions (warming) are still viable and important methods for thermoregulation assistance. However, in environments that are less stable and/or ideal, they do not have the same capacity to directly improve piglet temperature and induce sustained indirect effects on growth and survival. Our study supports previous findings in that the lightest piglets should be targeted for warming interventions, although benefits were seen in larger piglets.

## 5. Conclusions

Temperature regulation is critical for survival of piglets of all sizes, and it is important to continue working on ways to improve and assist in regulation, especially in low-birth-weight piglets. Injecting saline at 45 °C could be a useful strategy to warm piglets and indirectly increase colostrum intake; however, the temperature of the saline must be monitored closely and thus training would be required. Injecting saline and other suggested strategies such as drying piglets, in addition to measuring rectal temperature, is time sensitive and manually intensive. The priority should be on maintaining and optimizing the whole environment with specific interventions such as these, reserved for compromised and low-birth-weight piglets.

## Figures and Tables

**Table 1:** Mean ± SE for predicted colostrum intake (g) based on piglet weight change in 1589 piglet observations after bootstrapping at a root of 24. Body weight category: BWC1; ≤ 0.80 kg, BWC2; 0.81 kg to 1.10 kg, and BWC3; >1.10 kg. Saline = intraperitoneal 45 °C saline injection. N = number of piglet observations per group.

Treatment		RC1			
	N	BWC1 = 37	BWC2 = 49	BWC3 = 116	Overall
Control	130	66.9 ± 34.2	−439.7 ± 40.1 ^a^	76.86 ± 21.8 ^a^	−39.2 ± 26.7
Saline	72	189.0 ± 105.0	142.3 ± 33.4 ^b^	−135.9 ± 33.4 ^b^	−4.04 ± 29.6
		*RC2*			
	N	BWC1 = 0	BWC2 = 39	BWC3 = 234	
Control	154	-	295.2 ± 52.0	175.9 ± 16.6	183.9 ± 19.9
Saline	119	-	254.1 ± 39.9	214.6 ± 20.2	217.2 ± 17.1
		*RC3*			
	N	BWC1 = 68	BWC2 = 174	BWC3 = 872	
Control	491	166.0 ± 57.3	−177.8 ± 28.7 ^a^	237.4 ± 9.4 ^a^	205.3 ± 10.05
Saline	623	189.0 ± 55.0	246.8 ± 17.2 ^b^	351.0 ± 9.1 ^b^	326.9 ± 4.96
Overall		119.7 ± 3.98	99.97 ± 13.0	251.1 ± 5.99	Na

^a–b^ Denotes significance at *p* < 0.001 between treatments within BWC and RC.

**Table 2:** Mean ± SE for suckling weight gain (kg) from 24 h to 20 d in 1274 piglet observations after bootstrapping at a root of 24. Body weight category: BWC1; ≤0.80 kg, BWC2; 0.81 kg to 1.10 kg, and BWC3; >1.10 kg. Saline = intraperitoneal 45 °C saline injection. N = number of piglets per group.

		*Colostrum Intake* < 200 g			
	N	BWC1 = 36	BWC2 = 59	BWC3 = 115	**Overall**
Control	103	6.9 ± 0.37 ^a^	-	5.5 ± 0.35	6.24 ± 0.90
Saline	107	5.3 ± 0.37^b^	5.5 ± 0.35	5.6 ± 0.36	5.82 ± 0.05
		*Colostrum intake* ≥ 200 g			
	N	BWC1 = 0	BWC2 = 123	BWC3 = 941	
Control	451	-	5.8 ± 0.36 ^a^	4.7 ± 0.34	4.87 ± 0.03
Saline	613	-	6.5 ± 0.34	4.6 ± 0.33	5.02 ± 0.03
Overall		6.29 ± 0.07	6.13 ± 0.05	4.87 ± 0.02	

^a^ Denotes significance at *p* < 0.001 between treatment within BWC and colostrum intake group.

**Table 3:** Percentages and respective 95% confidence intervals of survival rates from day 1 (24 h) to 20 days for 1589 piglet observations after bootstrapping at a root of 24. Body weight category: BWC = 1; ≤0.80 kg, BWC = 2; 0.81 kg to 1.10 kg, and BWC = 3; >1.10 kg. Saline = intraperitoneal 45 °C saline injection. N = number of piglet observations included.

Parameter		N	BWC1 = 105	BWC2 = 262	BWC3 = 1222	Overall
Treatment	Control	753	11.7 (8.4–15.0)	74.4 (68.9–79.3)	72.5 (69.8–75.0)	73.3 (71.7–74.8)
	Saline	814	100 (95.0–100.0)	78.4 (74.8–81.6)	90.6 (89.3–91.8)	88.3 (87.2–89.4)
Sex	F	750	50.0 (43.2–100.0)	67.4 (62.5–72.0)	82.7 (80.6–84.6)	79.8 (78.3–81.2)
	M	839	100 (96.0–100.0)	83.6 (79.8–86.8)	84.2 (82.4–85.9)	81.6 (80.3–82.9)
Colostrum Intake	<200 g	335	100 (96.0– 100.0)	84.7 (80.3–88.2)	78.1 (74.7–81.2)	71.5 (69.0–74.0)
	≥200 g	1254	-	62.6 (61.5–69.6)	87.7 (86.7–88.6)	83.1 (82.0–84.1)
Overall			100 (95.0–100.0)	76.5 (73.0–79.6)	83.5 (81.9–84.9)	NA

**Table 4:** Percentages and respective 95% confidence intervals of survival rates at 20 d for 1589 piglet observations after bootstrapping at a root of 24. RC = 1 temperature ≤ 32.0 °C, RC = 2; temperature 32.1 °C to 35.0 °C, and C = 3; temperature ≥35.1 °C. Body weight categories; BWC1; ≤0.80 kg, BWC2; 0.81 kg to 1.10 kg, and BWC3; >1.10 kg. Saline = intraperitoneal 45 °C saline injection. N = number of piglet observations included.

Parameters		N	RC1 = 347	RC2 = 398	RC3 = 844	Overall
Body weight category	BWC1	105	13.78 (8.96–20.6)	-	-	52.3 (45.9–58.7)
	BWC2	262	39.2 (32.2–46.6)	62.6 (52.9–71.3)	80.5 (76.6–83.8)	72.1 (69.5–74.8)
	BWC3	1222	36.9 (31.3–42.8)	85.5 (825.3–88.2)	92.2 (91.0–93.3)	84.1 (83.0–85.1)
Treatment	Control	753	9.02 (0.01–14.2)	69.9 (64.6–74.8)	63.3 (48.9–75.7)	73.3 (71.7–74.8)
	Saline	814	100 (95.0–100.0)	83.8 (79.5–87.3)	92.7 (87.1–96.0)	88.3 (87.2–89.4)
Sex	F	750	20.8 (16.5–25.8)	75.8 (69.7–81.1)	100 (95.0–100.0)	79.8 (78.3–81.2)
	M	839	36.9 (30.9–43.3)	75.9 (70.1–80.9)	100 (95.0–100.0)	81.6 (80.3–82.9)
Colostrum intake	<200 g	335	44.2 (38.3–50.3)	71.0 (62.2–78.5)	100 (95.0–100.0)	71.5 (69.0–74.0)
	≥200 g	1254	16.2 (12.3–21.2)	80.1 (76.2–83.5)	100 (95.0–100.0)	83.1 (82.0–84.1)
Overall			30.6 (27.5–33.8)	81.3 (79.0–83.6)	90.4 (89.5–91.3)	NA

## Data Availability

Data available from the senior author on reasonable request.
